# Preexisting mild sleep disturbance as a vulnerability factor for inflammation-induced depressed mood: a human experimental study

**DOI:** 10.1038/tp.2016.23

**Published:** 2016-03-08

**Authors:** H J Cho, N I Eisenberger, R Olmstead, E C Breen, M R Irwin

**Affiliations:** 1 Cousins Center for Psychoneuroimmunology, Department of Psychiatry and Biobehavioral Sciences, Semel Institute for Neuroscience and Human Behavior, David Geffen School of Medicine, University of California Los Angeles, Los Angeles, CA, USA; 2 Department of Psychology, University of California Los Angeles, Los Angeles, CA, USA

## Abstract

Sleep disturbance and depression are common, particularly in females, and sleep disturbance is a well-known risk factor for depression. Systemic inflammation has been suggested as a potential mechanism of this association. This study examined whether preexisting sleep disturbance acted as a vulnerability factor for depressed mood induced by an inflammatory challenge in healthy females vs males. In a randomized double-blind placebo-controlled design, volunteers aged 18–50 (*N*=111; 67 females) were assigned to placebo or low-dose endotoxin. Before substance administration, sleep disturbance was assessed using the Pittsburgh Sleep Quality Index and dichotomized using median split (⩾3 vs <3). Self-reported depressed mood (profile of mood states) and circulating proinflammatory cytokines (interleukin-6, tumor necrosis factor-α) were repeatedly assessed over 6 h. Among females, moderation of depressed mood by sleep disturbance was significant even after adjustment for covariates (*X*^2^=12.73, df=6, *P*<0.05). There was a robust time-by-condition interaction in females with sleep disturbance (*X*^2^=26.22, df=6, *P*<0.001), but not in females without sleep disturbance (*X*^2^=8.65, df=6, *P*=0.19). Although cytokines increased equally in all females, the correlations between cytokines and depressed mood were significantly stronger in females with sleep disturbance. Among males, no moderating effect of sleep disturbance was observed. Inflammation-induced depressed mood was considerably more severe among females reporting mild sleep disturbance compared with those reporting no sleep disturbance, suggesting that even mild sleep disturbance may increase vulnerability for inflammation-induced depression in females. Furthermore, sleep disturbance appears to increase the vulnerability to depression by augmenting affective sensitivity to cytokines rather than by enhancing cytokine responses to inflammatory challenge in females.

## Introduction

Depression is a major public health burden because it is highly prevalent—the lifetime prevalence of major depression is almost 20% in the US general population^1^—and represents a leading cause of disability worldwide.^2^ Indeed, depression is projected to become the leading cause of disease burden by 2030.^3^ Thus, models for understanding and treating depression are critical. Although the dominant models of depression have focused on altered function in monoamine pathways, antidepressants—the mainstay of the current treatments for depression that target these pathways—typically only achieve remission rates of 30% or less when used as a monotherapy.^4^ This suboptimal effectiveness has led researchers to study biologically plausible models that would translate into new treatment and prevention strategies. Inflammation-induced depression is a promising alternative biological model of depression. Systemic inflammation is hypothesized to have an important role on the onset and perpetuation of some forms of depression.^5^

Sleep disturbance also has a substantial public health impact because approximately 25% of the US population report insomnia complaints^6^ and almost 10% fulfill diagnostic criteria for chronic insomnia.^7, 8, 9^ Sleep disturbance is a well-established risk factor for depression. A recent meta-analysis has demonstrated that sleep disturbance is an independent risk factor for depression.^10^ The mechanisms that underlie the association between sleep disturbance and depression are not yet known, but systemic inflammation has been suggested as a potential mechanism.^11^ Sleep disturbance leads to daytime increases in production and circulating levels of inflammatory cytokines.^12, 13, 14, 15^ We have further found that even modest amounts of sleep loss (that is, partial sleep deprivation) activate cellular and genomic markers of inflammation, in part, due to activation of cellular inflammatory signaling pathways (for example, nuclear factor-κB), especially in females.^16, 17, 18^

In addition, females appear to be especially vulnerable to the effects of an inflammatory challenge and show greater increases in feelings of social disconnection and depressed mood as compared with males.^19, 20, 21^ There are also well-known sex differences in the prevalence of inflammatory disorders, with females being twice as likely as males to develop autoimmune disorders including systemic lupus erythematosus, multiple sclerosis and rheumatoid arthritis.^22^ Although cardiovascular disease is thought to have an inflammatory basis and is more prevalent in males than females, recent epidemiologic data indicate that sleep disturbance is associated with a greater risk of cardiovascular disease in females than in males.^23, 24^ Finally, the female preponderance in the prevalence of both depression and sleep disturbance is a well-established finding.^8, 25^ Hence, there is substantial evidence that the interactive contribution of inflammatory states and sleep disturbance to mental and physical illness processes is far more significant in females.

Given the suggestion that systemic inflammation may be an underlying mechanism of the association between sleep disturbance and depression, particularly in females, we examined whether preexisting sleep disturbance enhanced the effect of an inflammatory challenge, endotoxin administration, on depressed mood among healthy females and males. Endotoxin administration is a safe procedure that, at low doses (<1 ng kg^−1^ of body weight), induces symptoms such as depressed mood, anhedonia, fatigue, reduced appetite and cognitive impairment, and has been used as a novel experimental paradigm of depression.^26^ We hypothesized that females, but not males, with sleep disturbance would exhibit greater increases in proinflammatory cytokines and depressed mood in response to endotoxin compared with those without sleep disturbance.

## Materials and methods

### Participants

One hundred and fifteen healthy participants (mean age 24.2±6.6 years; 69 females and 46 males) were deemed eligible and completed a randomized study of endotoxin administration (Supplementary Figure 1). Two female and two male participants who had missing data on sleep disturbance were removed from all analyses as sleep disturbance was the key variable of this study. Hence, the final analytical sample included 111 participants (mean age 24.1±6.6 years; 67 females and 44 males); 60 of them received endotoxin and 51 received placebo (subgroup comparisons shown in Table 1). Portions of these data have been previously reported in Moieni *et al.*^21^ The current study conducted an intention-to-treat analysis using all randomly assigned participants except for four participants who had missing data on sleep disturbance (hence *N*=111), whereas Moieni *et al.* excluded two participants for being outliers on the Beck Depression Inventory (BDI) and four participants for being outliers on sickness symptoms (hence *N*=109).

### Procedures

The study was conducted between March 2011 and August 2013 at the UCLA Clinical and Translational Research Center (CTRC) using a randomized, double-blind, placebo-controlled design. Briefly, each participant randomly received either low-dose endotoxin (0.8 ng kg^−1^ of body weight, *Escherichia coli* group O:113) or placebo (same volume of 0.9% saline) as an intravenous bolus. Endotoxin administration mimics the increases in inflammation that are found in inflammatory disorders, infections and also psychological stress.^27, 28, 29^ In the current study, endotoxin administration led to about 10-fold increase in interleukin-6 (IL-6) levels and fivefold increase in tumor necrosis factor-α (TNF-α) levels (by comparing geometric means), which may correspond to real-world clinical settings such as human immunodeficiency virus infection^27^ and rheumatoid arthritis.^30^ Blood samples (to assess circulating cytokine levels) were collected at baseline (T0) and then approximately every hour post injection for the next 6 h (T1–T6). The participants also completed hourly measures of mood and sickness symptoms.

### Behavioral assessments

#### Sleep disturbance

The Pittsburgh Sleep Quality Index (PSQI) was used to assess perceived sleep disturbance—evaluating components such as subjective sleep quality, sleep latency and use of sleep medications—during the preceding month.^31^ We used sleep disturbance as a binary variable using median split based on 111 participants (PSQI global score ⩾3 vs <3) throughout the study, hence indicating mild sleep disturbance. Clinical sleep impairment is identified by PSQI global score above 5.^31^

#### Depressed mood

Depressed mood was the primary outcome measure assessed from T0–T6 using the depression subscale of the short-form profile of mood states (POMS).^32, 33^ The participants rated the extent to which they felt at the moment (‘right now'): ‘unhappy', ‘sad', ‘blue', ‘hopeless', ‘discouraged', ‘miserable', ‘helpless' and ‘worthless' on a scale from 0 (not at all) to 4 (extremely). Depressed mood was calculated by summing scores from each of these items at each time point. The reliability of the scale (assessed at the time of peak response) was high (*α*=0.83). In addition, the participants completed the BDI^34^ at baseline before substance administration as a measure of depressive symptoms during the preceding week (‘the past week, including today'). Because this study evaluated preexisting sleep disturbance as the key variable (that is, moderator) and the baseline measure of depressive symptoms was used as a covariate in all analyses, depressive symptom scores were generated with the removal of the single sleep item from the original BDI. Rather than the baseline POMS, the baseline BDI was used as a covariate representing preexisting depressive symptoms because, while the POMS captures the snapshot of depressed mood at the present moment, the BDI assesses depressive symptoms experienced during the preceding week. Of note, although there was no significant difference in the baseline POMS between those with and without sleep disturbance (*t*-test *P*=0.32 in females; *P*=0.51 in males), the baseline BDI score was significantly higher in those with sleep disturbance (*P*-values <0.05). Higher levels of BDI scores, but not POMS scores, in those with sleep disturbance may be due to differences in symptom assessment between the two scales, the varying time frame (past week for the BDI and present moment for the POMS) or both. As compared with those without sleep disturbance, those with sleep disturbance showed significantly higher scores on the following BDI items: self-dissatisfaction, self-dislike, self-accusation and indecisiveness. None of these items were explicitly evaluated by the POMS, with a possible exception of self-dislike, which has some conceptual overlap with ‘worthless'.

#### Self-reported sickness symptoms

Physical sickness symptoms (headaches, muscle pain, shivering, nausea, breathing difficulties and fatigue) were assessed from T0 to T6. The participants rated the extent to which they felt each symptom on a scale from 0 (no symptoms) to 4 (very severe symptoms). Scores from each of these items were then dichotomized for each time point: any reports of 2 (‘moderate') or greater for any of the symptoms were scored as a physical symptom response, zero otherwise.

### Plasma levels of cytokines

Plasma levels of IL-6 and TNF-α were quantified by high sensitivity bead-based multiplex (Luminex) immunoassays (Performance High Sensitivity Human Cytokine, R&D Systems, Minneapolis, MN, USA), as previously described.^21^

### Statistical analysis

Given the repeated nature of measurement, linear regression analysis using mixed-effects model was implemented to assess: the effect of endotoxin (vs placebo) on the temporal changes of depressed mood and cytokines from baseline throughout 6 h of the study procedure; as well as the moderation of this effect by sleep disturbance. Mixed-effects regression models use all the available data during the study procedure, can properly account for correlation between repeated measurements on the same subject, have greater flexibility to model time effects and can handle missing data more appropriately than traditional models such as repeated-measures analysis of variance.^35^ Because there was an important sex difference in changes of depressed mood following endotoxin administration as previously reported,^21^ all analyses were conducted separately in females and males. Because the cytokine values were not normally distributed, values were natural log-transformed. As the moderation by sleep disturbance was the focus of this investigation, and sleep disturbance was an observed variable without a random allocation, all analyses were adjusted for age, race, body mass index and baseline depressive symptoms as assessed by the BDI without the sleep item. In particular, although all participants were free of depressive disorders as ascertained by the Structured Clinical Interview for DSM Disorders,^36^ those with sleep disturbance reported higher levels of depressive symptoms at baseline as assessed using the BDI without the sleep item (see ‘Results' section). For moderation tests, mixed-effects regression models included the three-way interaction term among condition, time and sleep disturbance (condition-by-time-by-sleep disturbance). Subsequently, subgroup analyses stratified by sleep disturbance were conducted in the same format to assess the actual difference in the effects of endotoxin according to the presence or absence of sleep disturbance. Last, the correlations between cytokine levels and depressed mood within the endotoxin group were also examined using mixed-effects linear regression. The analyses were conducted using Stata 14 (StataCorp, College Station, TX, USA) and statistical significance was established at *P*⩽0.05 (two-sided). See Supplementary Methods for more details.

## Results

### Baseline sample characteristics

As described in the ‘Methods' section, the final analytical sample included 111 participants. When those with and without sleep disturbance (PSQI global score ⩾3 vs <3) were compared separately among females and males as presented in Table 1 using *t*-tests or chi-square tests, age, race composition and body mass index were not significantly different. Based on the BDI without the sleep item, females and males with sleep disturbance were significantly more depressed compared with their counterparts without sleep disturbance (*P*-values <0.05).

### Depressed mood changes in response to endotoxin and moderation by sleep disturbance

As previously reported,^21^ there was an important sex difference in changes of depressed mood following endotoxin; females experienced robust increases in depressed mood in response to endotoxin, whereas males exhibited no significant changes in depressed mood (adjusted *X*^2^ for condition × time × sex interaction=11.90, df=6, *P*=0.06 (adjusted for age, race, body mass index and baseline depressive symptoms)). Given this overall sex difference, moderation of depressed mood changes by sleep disturbance was tested separately in females and males. We adopted this approach because there are well-established sex differences in inflammatory disorders, sleep disturbance and depression;^8, 22, 25^ and our previous endotoxin study has also demonstrated important sex differences.^19, 20^

**Figure 1:**
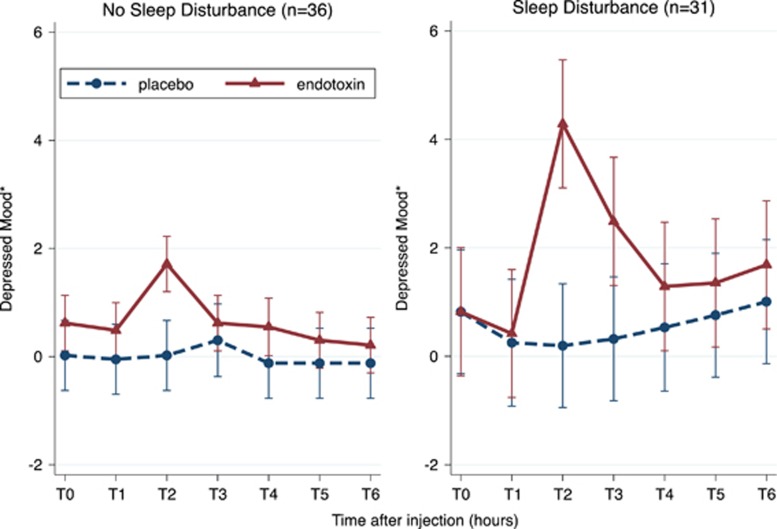
Effect of endotoxin on depressed mood over time in females according to sleep disturbance. Depressed mood was assessed at baseline (T0) and then approximately every hour after injection for the next 6 h (T1–T6). T2 was assessed at 1 h and 40 min after injection; T3 was assessed at 3 h and 30 min after injection; and T4–T6 were assessed hourly after T3. Error bars indicate 95% confidence intervals. *Marginal means adjusted for age, race, BMI and baseline depressive symptoms. BMI, body mass index.

In females, we tested the hypothesis that those with sleep disturbance would experience greater increases in depressed mood in response to endotoxin as compared with those without sleep disturbance. As shown in Figure 1, there was a significant difference in the effects of endotoxin on depressed mood according to sleep disturbance; that is, there was a significant moderation by sleep disturbance (adjusted *X*^2^ for condition × time × sleep disturbance interaction=12.73, df=6, *P*<0.05). According to the analyses at each time point, condition × time × sleep disturbance interaction was significant at T2 (adjusted *B*=3.00, *P*<0.01) and T3 (adjusted *B*=2.46, *P*<0.05). According to analyses stratified by sleep disturbance, there was a robust condition × time interaction in the female subgroup with sleep disturbance (adjusted *X*^2^=26.22, df=6, *P*<0.001). However, in the female subgroup without sleep disturbance, there was no significant time × condition interaction (adjusted *X*^2^=8.65, df=6, *P*=0.19). We then performed a *post hoc* analysis to test which domain of the PSQI was responsible for this moderation, using the previously validated three-factor structure of the PSQI: sleep efficiency, perceived sleep quality and daily disturbances.^37^ Daily disturbances (a factor composed of the PSQI components sleep disturbance and daytime dysfunction), but not sleep efficiency or perceived sleep quality, significantly moderated the effect of endotoxin on depressed mood among females (adjusted *X*^2^ for condition × time × daily disturbances interaction=18.48, df=6, *P*=0.005).

In addition, to examine whether the moderation of depressed mood changes by sleep disturbance was independent from sickness symptoms, we conducted an exploratory analysis where the moderation by sleep disturbance in females was further adjusted for physical sickness symptoms (for example, fatigue and pain) throughout all time points T0–T6. Thus, sickness symptoms were added to the condition × time × sleep disturbance interaction model in their longitudinal format. Despite this highly conservative approach (as fatigue is one of the diagnostic criteria for major depressive disorder), the moderation by sleep disturbance in females was practically unchanged (adjusted *X*^2^=12.26, df=6, *P*=0.056 (compared with 12.73, df=6, *P*<0.05 before adjusting for sickness symptoms)). According to the analyses at each time point, condition × time × sleep disturbance interaction was again significant at T2 (adjusted *B*=2.96, *P*<0.01) and at T3 (adjusted *B*=2.44, *P*<0.05). Following this exploratory analysis, we also examined whether females with sleep disturbance showed greater increases in sickness symptoms in response to endotoxin, testing sickness symptoms as an outcome and sleep disturbance as a moderator. There was no difference in the effect of endotoxin on sickness symptoms according to sleep disturbance, that is, no significant condition × time × sleep disturbance interaction (adjusted *X*^2^=4.48, df=6, *P*=0.61).

In males, moderation of depressed mood changes by sleep disturbance was similarly tested. As hypothesized, there was no difference in the effects of endotoxin on depressed mood according to sleep disturbance; that is, there was no significant moderation by sleep disturbance (adjusted *X*^2^ for condition × time × sleep disturbance interaction=4.74, df=6, *P*=0.58). In the male subgroup with sleep disturbance, there was no significant condition × time interaction (adjusted *X*^2^=3.99, df=6, *P*=0.68); and likewise in the male subgroup without sleep disturbance (adjusted *X*^2^=9.69, df=6, *P*=0.14).

### Inflammatory responses to endotoxin and sleep disturbance in females

**Figure 2:**
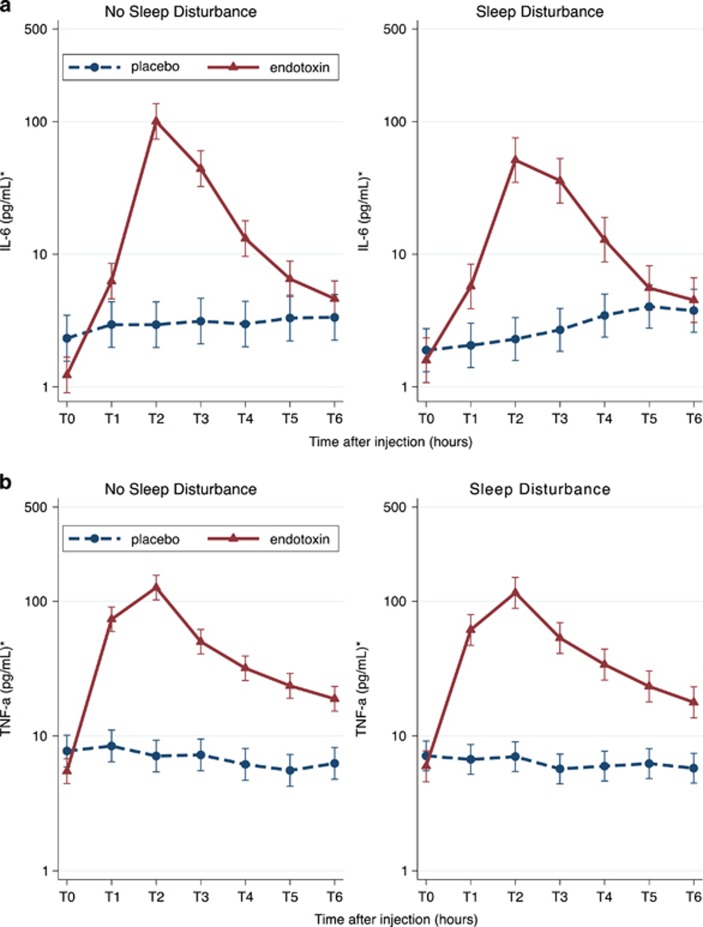
Effect of endotoxin on cytokines ((**a**) IL-6; (**b**) TNF-α) over time in females according to sleep disturbance. Cytokines were measured at baseline (T0) and then approximately every hour after injection for the next 6 h (T1–T6). T2 was assessed at 1 h and 40 min after injection; T3 was assessed at 3 h and 30 min after injection; and T4–T6 were assessed hourly after T3. Error bars indicate 95% confidence intervals. *Marginal means adjusted for age, race, BMI and baseline depressive symptoms. BMI, body mass index; IL, interleukin; TNF, tumor necrosis factor.

To understand whether differences in inflammatory responses accounted for the greater increase in depressed mood in females with sleep disturbance as compared with females without sleep disturbance, we tested moderation of inflammatory responses to endotoxin by sleep disturbance (that is, changes in IL-6 and TNF-α levels). In contrast to our initial hypothesis that females with sleep disturbance would exhibit greater increases in proinflammatory cytokines in response to endotoxin, there were no differences in the effects of endotoxin on IL-6 or TNF-α levels according to sleep disturbance (Figure 2); that is, condition × time × sleep disturbance interactions were not significant (adjusted *X*^2^=7.11, df=6, *P*=0.31 for IL-6; adjusted *X*^2^=4.73, df=6, *P*=0.58 for TNF-α).

### Relationships between cytokines and depressed mood and sleep disturbance in females

**Figure 3:**
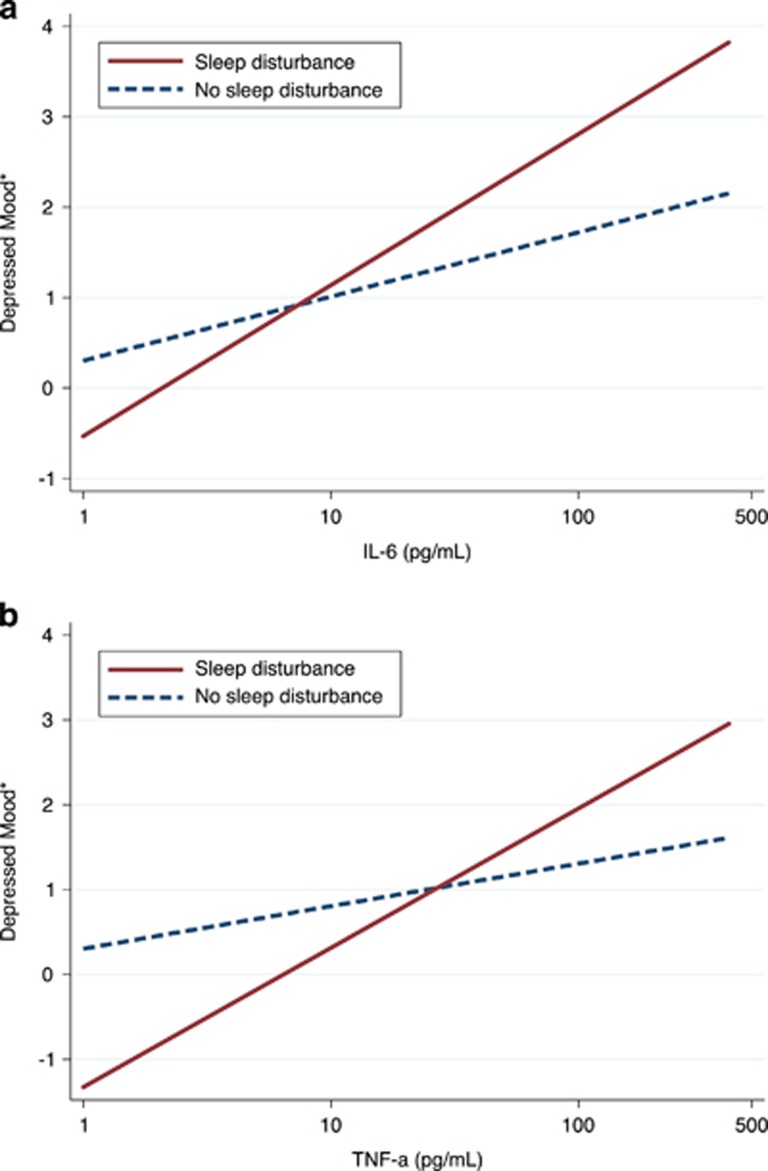
Correlations between cytokines ((**a**) IL-6; (**b**) TNF-α) and depressed mood in females according to sleep disturbance. Correlations represent adjusted linear predictions estimated using mixed-effects model. *Marginal means adjusted for age, race, BMI and baseline depressive symptoms. BMI, body mass index; IL, interleukin; TNF, tumor necrosis factor.

As there were no differences in cytokine levels according to the presence or absence of sleep disturbances in females, we examined whether the affective sensitivity to cytokines was differentially heightened in females with sleep disturbance vs females without sleep disturbance. For this purpose, correlations between cytokines and depressed mood were examined in females exposed to endotoxin, using mixed-effects model linear regression. Accordingly, cytokines and depressed mood were entered, respectively, as independent and dependent variables in the models in their longitudinal format, thus reflecting all the measurements performed during the study. Correlations between cytokines and depressed mood were significantly stronger in females with sleep disturbance compared with females without sleep disturbance (Figure 3); that is, there were significant moderations by sleep disturbance (adjusted *B* for IL-6 × sleep disturbance interaction=0.42, *P*<0.05; adjusted *B* for TNF-α × sleep disturbance interaction=0.50, *P*=0.05). Correlations were more than twice as strong in females with sleep disturbance (adjusted *B*=0.67, *P*<0.001 for IL-6; adjusted *B*=0.69, *P*<0.01 for TNF-α) as in females without sleep disturbance (adjusted *B*=0.31, *P*<0.001 for IL-6; adjusted *B*=0.27, *P*<0.05 for TNF-α).

## Discussion

This study provides experimental evidence to support the hypothesis that self-reported sleep disturbance is a vulnerability factor for inflammation-induced depressed mood in females—but not in males—using an experimental model that mimics increased inflammation found in infections,^27^ autoimmune diseases^29^ and also psychological stress.^28^ When exposed to an inflammatory challenge (that is, endotoxin), females who reported just mildly disturbed sleep experienced exaggerated increases in depressed mood as compared with women who reported no sleep disturbance. Furthermore, whereas circulating proinflammatory cytokines increased equally in females with and without sleep disturbance, cytokine levels were more strongly correlated with depressed mood in females with sleep disturbance. However, none of these moderating effects of sleep disturbance were observed in males, in part, due to the fact that males do not show significant changes in depressed mood following endotoxin challenge. Taken together, these results suggest that sleep disturbance may alter affective sensitivity to inflammatory challenge in females, but not in males. Females appear to be more sensitive to the affective consequences of inflammation, which may, in part, explain the increased likelihood for females to develop depressive disorders, especially among those who have sleep disturbance.

Although the current study did not find a significant endotoxin effect on mood in males, previous studies have reported endotoxin-induced depressed mood in males (reviewed in Schedlowski *et al.*^38^). However, it should be noted that the majority of those studies have been conducted in male-only sample, hence not allowing for a direct comparison across sexes (sex differences discussed in more detail elsewhere^21^).

Although systemic inflammation has been suggested as a potential mechanism of the well-known association between sleep disturbance and depression,^11^ to date, no experimental studies have examined the complex inter-relationships between sleep disturbance, inflammation and depression. In line with the current findings, an observational study has previously shown that self-reported sleep disturbance predicted the onset of depression during interferon-α treatment, which elicits a heightened inflammatory state.^39^ The current study extends this observational evidence by examining the inter-relationships between sleep disturbance, inflammation and depression in a highly controlled experimental design.

The literature on the relationship between sleep and innate immunity (reviewed in Zielinski and Krueger^40^ and Irwin *et al.*^15^) supports the role of sleep disturbance in heightened systemic inflammation, which may explain the contribution of sleep disturbance to the risk of inflammatory diseases and depressive disorders including inflammation-induced depression. The current study offers further insight into how sleep disturbance would increase depression risk. The findings of this study provide evidence in support of a ‘two hit' model of depression (with the caveat that this model may apply to females only). In the present study, this two-hit model posits that sleep disturbance serves as a vulnerability factor; in turn, subsequent exposure to heightened inflammatory states such as an infectious challenge or psychological stress triggers increases in depressive symptoms (Supplementary Figure 2). Alternatively, given the evidence that experimentally induced sleep disturbance induces greater increases in depressive symptoms in persons with an inflammatory disorder,^41^ inflammation might serve as a vulnerability factor, and subsequent exposure to sleep disturbance might trigger increases in depressive symptoms. The latter iteration of a two-hit model of depression requires further testing. Moreover, sleep disturbance increased the vulnerability to depressed mood by augmenting affective sensitivity to cytokines rather than by enhancing the magnitude of cytokine responses to endotoxin. Indeed, stronger correlations between cytokine levels and depressed mood were found in females with sleep disturbance as compared with females without sleep disturbance. The mechanisms whereby sleep disturbance primes affective sensitivity to proinflammatory cytokines have yet to be elucidated. Although beyond the scope of this manuscript, additional analyses are evaluating the contribution of other pro- and anti-inflammatory cytokines on behavioral and neural responses to endotoxin, as well as the relation of cytokine gene polymorphisms, tryptophan metabolism and glucocorticoid resistance in moderating such responses.

Defining vulnerability factors for inflammation-induced depression have practical implications for developing and implementing prevention strategies for depression, which should include identification of at-risk individuals and actual interventions. For example, a prospective cohort study has found that adolescent females with childhood adversity were more vulnerable to the depressogenic effects of endogenous IL-6 compared with those without childhood adversity.^42^ On the basis of the current data, females with two hits, sleep disturbance and inflammation, would represent a high-risk group to be prioritized for depression prevention efforts using interventions that target sleep disturbance or inflammation. If corroborated by future translational studies, the current study may have important clinical implications for depression prevention.

Several limitations should be considered. First, although this experimental model of depression by endotoxin administration is a unique opportunity for studying depression and has been used in several previous studies,^26^ endotoxin-induced depressed mood in healthy volunteers cannot be equated with clinical depression. Future clinical studies will be required to corroborate and translate the current findings into clinical practice. Second, a related concern would be the possibility that depressed mood in this experimental study could have been majorly influenced by physical sickness symptoms, although in clinical practice depressed individuals experience both emotional and somatic symptoms of depression in varying degrees. To minimize this possibility, we used a depression scale that only assesses emotional symptoms such as ‘unhappy', ‘sad' and ‘hopeless'. Furthermore, both the main effect of endotoxin on depressed mood (reported elsewhere^21^) and the moderation of depressed mood by sleep disturbance were independent from physical sickness symptoms; and the effects of endotoxin on physical sickness symptoms were not moderated by either sex (reported elsewhere^21^) or sleep disturbance. Third, although this was a relatively large experimental study, owing to the analyses using subgroups by gender and sleep disturbance, each subgroup was small and some of the nonsignificant findings could be due to low power. However, it should be noted that, despite this potential problem of power, the present study revealed some notable significant findings. Fourth, the assessment of sleep disturbance relied on self-report, whereas polysomnography is arguably the objective standard to assess insomnia. However, assessment of sleep problems in clinical practice primarily relies on patients' subjective evaluation. Fifth, although this was a highly controlled experimental study, the main variable of interest, sleep disturbance, was an observed variable and not a randomly assigned experimental variable, hence limiting causal inferences from the current findings. To remedy this limitation as much as possible, all analyses were adjusted for potential confounders such as age, race, body mass index and baseline depressive symptoms. Sixth, menstrual cycle data were not collected and thus we could not examine whether menstrual cycle was associated with changes in mood or inflammation.

In summary, inflammation-induced depressed mood was considerably more severe among healthy females reporting mild sleep disturbance compared with those reporting no sleep disturbance, suggesting that even mild sleep disturbance may increase the vulnerability for inflammation-induced depression in females. Furthermore, sleep disturbance appears to increase the vulnerability to depression by augmenting affective sensitivity to proinflammatory cytokines rather than by enhancing cytokine responses to an inflammatory challenge in females. Of note, these moderating effects of sleep disturbance were not observed in males. The current findings provide insight into the mechanisms underlying the association between sleep disturbance and depression, and, if corroborated by future translational studies, may have important clinical implications for depression prevention.

## Figures and Tables

**Table 1:** Sample characteristics at baseline

*Characteristic*	*Females with sleep disturbance (*n=*31)*	*Females without sleep disturbance (*n=*36)*	P^a^	*Males with sleep disturbance (*n=*19)*	*Males without sleep disturbance (*n=*25)*	P^a^
Assigned to endotoxin (%)	15 (48.4)	22 (61.1)	0.30	9 (47.4)	14 (56.0)	0.57
Mean age (s.d.)	23.5 (6.3)	23.0 (6.4)	0.74	24.5 (5.9)	26.1 (7.6)	0.44
Mean body mass index (s.d.)	22.9 (2.9)	24.1 (2.8)	0.09	24.7 (2.7)	24.0 (2.3)	0.39
						
*Race (%)*						
Caucasian	11 (35.5)	13 (36.1)		8 (42.1)	11 (44.0)	
Asian/Pacific Islander	6 (19.4)	9 (25.0)	0.69	8 (42.1)	8 (32.0)	0.85
African American	1 (3.2)	2 (5.6)		0 (0.0)	1 (4.0)	
Latino	8 (25.8)	10 (27.8)		2 (10.5)	4 (16.0)	
Other	5 (16.1)	2 (5.6)		1 (5.3)	1 (4.0)	
						
Mean depression score (s.d.)^b^	4.3 (5.0)	2.0 (3.0)	<0.05	2.9 (2.7)	1.4 (2.1)	<0.05	
Mean PSQI global score (s.d.)	4.5 (1.5)	1.1 (0.8)	<0.0001	4.0 (1.0)	1.3 (0.6)	<0.0001	

Abbreviation: PSQI, Pittsburgh Sleep Quality Index.

a *P*-values from *t*-tests when the sample characteristic is a continuous variable and from chi-square tests when the sample characteristic is a categorical variable.

b Depressive symptoms assessed using the Beck Depression Inventory total score without the sleep item.
