# Supplementary material for: Preexisting mild sleep disturbance as a vulnerability factor for inflammation-induced depressed mood: a human experimental study
Source: Transl Psychiatry. 2016 Mar 8;6(3):e750–. doi: 10.1038/tp.2016.23 (PMC4872448; doi:10.1038/tp.2016.23)
Supplement: Supplementary Figures [file tp201623x1.docx]

**Supplemental Figure 1. CONSORT Diagram**

Allocated to endotoxin condition (n=66)

- Received endotoxin (n=61)
- Did not receive endotoxin (n=5)
- subject cancelled (n=1)
- difficulty drawing blood (n=1)
- lost consciousness prior to receiving drug (n=1)
- failed drug test prior to session (n=2)

Allocated to placebo condition (n= 57)

- Received placebo (n=54)
- Did not receive placebo (n=3)
- subject cancelled (n=3)

Excluded (n=1993)

- Not meeting inclusion criteria (n=1967)
- Declined to participate (n=15)
- Other reasons (n=11)

Assessed for eligibility (n= 2116)

## Analysis (n=111)

## Allocation (n=115)

Randomized (n=123)

## Enrollment

Analysed (n=60)

Excluded from analysis (n=1)

- missing key variable, i.e., sleep disturbance (n=1)

Analysed (n=51)

Excluded from analysis (n=3)

- missing key variable, i.e., sleep disturbance (n=3)


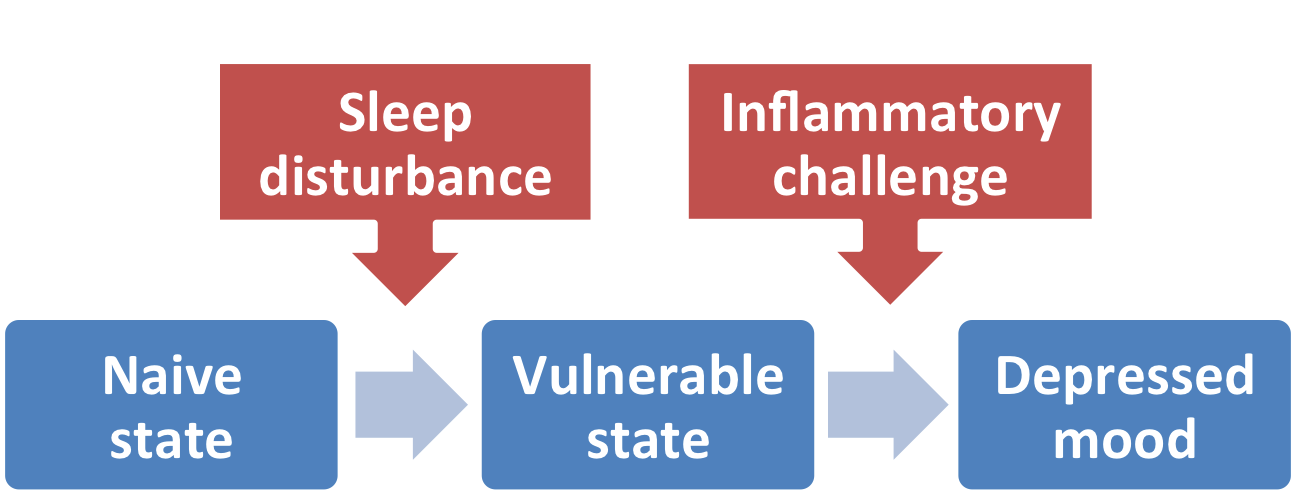


**Supplemental Figure 2. Proposed ‘two hit’ model of depression involving sleep disturbance and inflammatory challenge**
