# Supplementary material for: Preexisting mild sleep disturbance as a vulnerability factor for inflammation-induced depressed mood: a human experimental study
Source: Transl Psychiatry. 2016 Mar 8;6(3):e750–. doi: 10.1038/tp.2016.23 (PMC4872448; doi:10.1038/tp.2016.23)
Supplement: Supplementary Information [file tp201623x2.docx]

**SUPPLEMENTAL METHODS**

**Participants & Eligibility Assessment**

One hundred and fifteen healthy participants were deemed eligible and completed a randomized study of endotoxin administration (ClinicalTrials.gov NCT01671150), as described elsewhere in detail.^1^ Interested participants were screened for eligibility by a structured telephone interview and an in-person screening session. Exclusion criteria included: presence of comorbid medical conditions; prescription medication use; an Axis I psychiatric disorder based on the Structured Clinical Interview for DSM Disorders (SCID)^2^; body-mass index (BMI) greater than 30; recent nightshift work or time zone shifts greater than 3 hours; evidence of drug use from a positive urine test; a positive pregnancy test; abnormalities on the screening laboratory tests.

**Randomization**

A statistician generated the randomization list using a computer-generated randomization table. Except for this statistician and a research pharmacist who prepared endotoxin and placebo, all participants, investigators, and study staff were blind to allocation. Endotoxin and placebo were prepared to appear identical in volume, weight, and color.

**Procedures**

Catheters with a heparin lock were placed into the dominant forearm (right) for hourly blood draws and into the non-dominant forearm (left) for a continuous saline flush and drug administration. Each participant randomly received either low-dose endotoxin (0.8 ng/kg of body weight, *E. coli* group O:113: BB-IND 12948 to M.R.I.) or placebo (same volume of 0.9% saline) as an intravenous bolus. The endotoxin was provided by the National Institutes of Health Clinical Center;^3^ previous research has demonstrated the safety of this reference endotoxin across many different samples.^4, 5^ Blood samples were collected at baseline (T0) and then approximately every hour post-injection for the next six hours (T1-T6). Participants also completed hourly measures of mood and sickness symptoms. Because participants completed a neuroimaging session (reported separately) starting at exactly 2 hours post-injection, T2 was assessed prior to this scanning session at 1 hour 40 minutes post-injection and T3 was assessed immediately after the scan at 3 hours and 30 minutes post-injection; T4-T6 were assessed hourly after T3. Participants were discharged from the UCLA Clinical and Translational Research Center (CTRC) following the last blood draw once self-reported physical and psychological symptoms returned to baseline levels; participants were thanked, debriefed, and paid for their participation (US $220 for screening and the day of the study). All subjects provided written consent before participating. All procedures were approved by UCLA Human Subjects Protection Committee.

**Cytokine Assays**

Whole blood samples were collected in pre-chilled EDTA tubes, centrifuged at 4°C, and aliquots of plasma stored at -70°C until assayed. Plasma levels of IL-6 and TNF-α were quantified by high sensitivity bead-based multiplex (Luminex) immunoassays (Performance High Sensitivity Human Cytokine, R&D Systems, Minneapolis, MN), as previously described.^1^ All samples from each subject (baseline and all subsequent timepoints) were assayed on the same 96-well plate; multiplex assays were chosen for the analyses because of the large dynamic range necessary to evaluate both low physiologic (baseline) and very high (post-endotoxin) cytokine concentrations in the same assay. The ranges of detection for IL-6 and TNF-α were 0.2-3800 pg/mL and 0.8-3100 pg/mL, respectively, and no samples exceeded the upper limit of detection for either analyte. The mean intra-assay coefficient of variation of the standards was <8% for IL-6 and TNF-α; the inter-assay coefficient of variation of an internal laboratory quality control sample was ≤13% for both analytes. Every subject demonstrated the expected profile of change of cytokine concentrations over time, based on previous studies.^6, 7^

**Unexpected Adverse Effects**

Of the 115 participants that completed the study, five participants experienced unexpected, but not harmful, symptoms (e.g., diarrhea, nausea) that prevented them from completing all components of the study protocol (i.e., the aforementioned neuroimaging component). An additional participant lost consciousness prior to drug administration upon insertion of the intravenous catheter; this participant did not complete the study due to safety concerns.

**Sample Size Calculation**

Based on our previous endotoxin study,^7^ which showed a robust effect of endotoxin vs. placebo on depressed mood (Cohen’s d=0.73), a sample size of 100 (i.e., 50 per condition) would yield greater than 90% power (α=0.05, two-tailed). This relatively large sample was also required to obtain an adequate though more modest level of 80% power for the estimation of potential sex differences in the effects of endotoxin.

**Data Analysis**

Given the repeated nature of measurement, linear regression analysis using mixed-effects model was implemented. Mixed-effects regression models included depressed mood (or cytokines) as the dependent variable; and condition (endotoxin vs. placebo), time, and an interaction term as the independent variables. The interaction term was either “condition-by-time”, “condition-by-time-by-sex”, or “condition-by-time-by-sleep disturbance” depending on the question being tested. Note that the dependent variables (depressed mood and cytokines) were included in the analyses in their longitudinal format – i.e., they reflected all the measurements performed during the study – in order to take into account not only these variables at individual timepoints but also their evolution across time. Lastly, the correlations between cytokine levels and depressed mood within the endotoxin group were examined also using mixed-effects linear regression by including these variables in in their longitudinal format: first formally testing the moderation of the correlation between cytokines and depressed mood by sleep disturbance; and then conducting subgroup analyses stratified by sleep disturbance.

**References**

1. Moieni M, Irwin MR, Jevtic I, Olmstead R, Breen EC, Eisenberger NI. Sex Differences in Depressive and Socioemotional Responses to an Inflammatory Challenge: Implications for Sex Differences in Depression. *Neuropsychopharmacology* 2015.

2. First MB, Gibbon M. *User's guide for the structured clinical interview for DSM-IV axis I disorders: SCID-1 clinician version*. American Psychiatric Pub1997.

3. Suffredini AF, Fantuzzi G, Badolato R, Oppenheim JJ, O'Grady NP. New insights into the biology of the acute phase response. *J Clin Immunol* 1999; **19**(4)**:** 203-214.

4. Suffredini A, O’Grady N, Brade H, Opal S, Vogel S, Morrison D. Pathophysiological responses to endotoxin in humans. *Endotoxin in health and disease* 1999**:** 817-830.

5. Andreasen A, Krabbe K, Krogh-Madsen R, Taudorf S, Pedersen B, Moller K. Human endotoxemia as a model of systemic inflammation. *Curr Med Chem* 2008; **15**(17)**:** 1697-1705.

6. Eisenberger NI, Inagaki TK, Mashal NM, Irwin MR. Inflammation and social experience: an inflammatory challenge induces feelings of social disconnection in addition to depressed mood. *Brain Behav Immun* 2010; **24**(4)**:** 558-563.

7. Eisenberger NI, Inagaki TK, Rameson LT, Mashal NM, Irwin MR. An fMRI study of cytokine-induced depressed mood and social pain: the role of sex differences. *NeuroImage* 2009; **47**(3)**:** 881-890.
